# Supplementary material for: Trends in Prostate Cancer Incidence and Survival by Gleason Score from 2000 to 2020: A Population-Based Study in Northeastern Italy
Source: Curr Oncol. 2025 Jul 29;32(8):426. doi: 10.3390/curroncol32080426 (PMC12384114; doi:10.3390/curroncol32080426)
Supplement: Supplementary file 1 [file curroncol-32-00426-s001.zip › curroncol-3749934-supplementary.pdf]

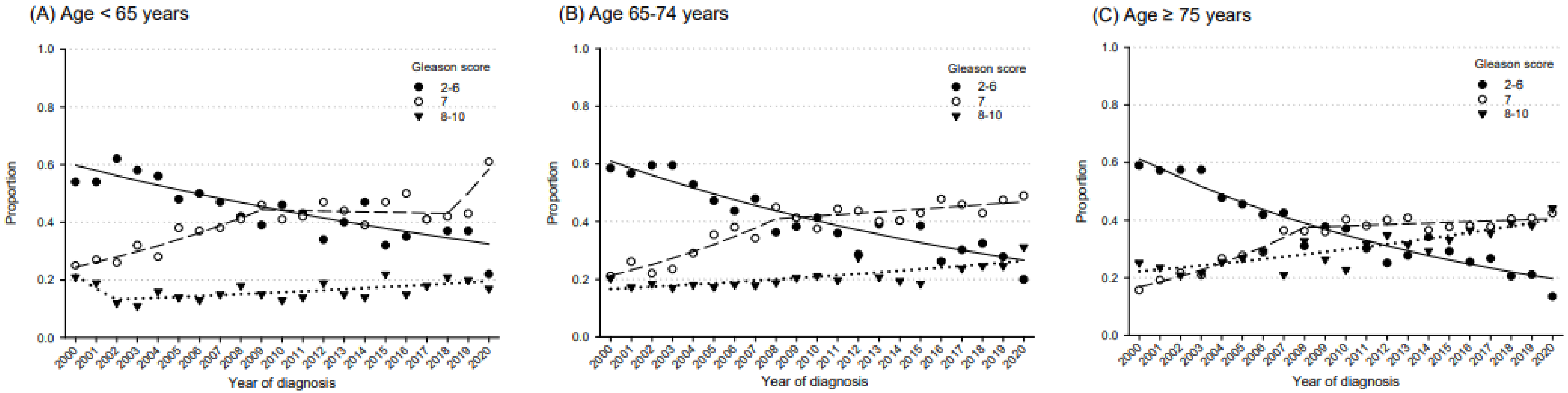

Gleason	Trend 1 Years	Trend 1 APC	Trend 2 Years	Trend 2 APC	Trend 3 Years	Trend 3 APC
2-6	2000-2020	-3.0*				
7	2000-2009	+6.8*	2009-2018	-0.3	2018-2020	+16.6*
8-10	2000-2002	-22.0	2002-2020	+2.2		

Gleason	Trend 1 Years	Trend 1 APC	Trend 2 Years	Trend 2 APC
2-6	2000-2020	-4.1*		
7	2000-2008	+8.5*	2008-2020	+1.1
8-10	2000-2020	+2.3*		

Trend 1 Years	Trend 1 APC	Trend 2 Years	Trend 2 APC
2000-2020	-5.5*		
2000-2008	+10.6*	2008-2020	+0.6
2000-2020	+3.0*		

Supplementary Figure S1. Trends in proportion of prostate cancer (PCa) cases by Gleason score and age group, with corresponding joinpoint analyses: (A) Age <65 years; (B) Age 65-74 years; (C) Age ≥75 years. Friuli Venezia Giulia, Italy, 2000-2020.

The data points represent observed proportions of cases, while the lines illustrate the trends modeled by joinpoint regression analysis. For each segment, the Annual Percent Change (APC) is calculated and presented in the accompanying table, with the respective trend segments identified.
